# Complete genome sequence of *Liberibacter crescens* BT-1

**DOI:** 10.4056/sigs.3326772

**Published:** 2012-12-12

**Authors:** Michael T. Leonard, Jennie R. Fagen, Austin G. Davis-Richardson, Michael J. Davis, Eric W. Triplett

**Affiliations:** 1 Microbiology and Cell Science Department, Institute of Food and Agricultural Sciences, University of Florida, Gainesville, FL, USA; 2 Plant Pathology Department, Citrus Research and Education Center, Institute of Food and Agricultural Sciences, Lake Alfred, FL, USA

**Keywords:** Huanglongbing, citrus greening, reduced genome, fastidious, hybrid assembly

## Abstract

*Liberibacter crescens* BT-1, a Gram-negative, rod-shaped bacterial isolate, was previously recovered from mountain papaya to gain insight on Huanglongbing (HLB) and Zebra Chip (ZC) diseases. The genome of BT-1 was sequenced at the Interdisciplinary Center for Biotechnology Research (ICBR) at the University of Florida. A finished assembly and annotation yielded one chromosome with a length of 1,504,659 bp and a G+C content of 35.4%. Comparison to other species in the *Liberibacter* genus, *L. crescens* has many more genes in thiamine and essential amino acid biosynthesis. This likely explains why *L. crescens* BT-1 is culturable while the known *Liberibacter* strains have not yet been cultured. Similar to *Candidatus* L. asiaticus psy62, the *L. crescens* BT-1 genome contains two prophage regions.

## Introduction

Huanglongbing (HLB), also known as citrus greening, is a disease that poses a major economic threat to the worldwide citrus industry [1,2]. The disease was discovered to be present in Florida in 2005 and is characterized by yellowing of citrus tree leaves, premature defoliation, small bitter fruit, and a pale green fruit color after ripening. No known cure for the disease has been discovered, but preventative measures include chemical treatment against insect vectors and removal of infected trees to prevent the spread of disease [3,4].

The causal agents of HLB are believed to be *Candidatus* Liberibacter asiaticus, *Candidatus* L. africanus, and *Candidatus* L. americanus, named according to the regions of where the organism was first identified [5,6]. Similar diseases have been found to occur in potatoes (*Solanum tuberosum*) and other solanaceous crops infected with *Candidatus* L. solanacearum [7]. Additionally, the *Liberibacter* genus contains the plant endophyte *Candidatus* L. europaeus [8], signifying that virulence in the *Liberibacter* genus is not universal. None of these organisms have been cultured but a metagenomic analysis of phloem suggests that this is the only bacterium present in the phloem of symptomatic trees [9].

Due to the highly fastidious nature of the genus *Liberibacter*, research on these organisms has traditionally been limited to electron microscopy and genomic analysis [3,7,10]. However, one species of the genus, *Liberibacter crescens*, has recently been cultured and characterized [11], and the relationship between its genome and close relatives will be the focus here.

In order to gain insight on both the virulence and metabolism of the genus *Liberibacter*, all available genomes of the *Liberibacter* spp. were compared to *Liberibacter crescens*. To date, the genomes of *Candidatus* L. asiaticus and *Candidatus* L. solanacearum are publicly available. The differences between these species may be responsible for the fastidious nature of the *Liberibacter* spp. Sequencing, assembly, and annotation of *L. crescens* were performed in order to proceed with the investigation.

## Classification and features

**Figure 1:**
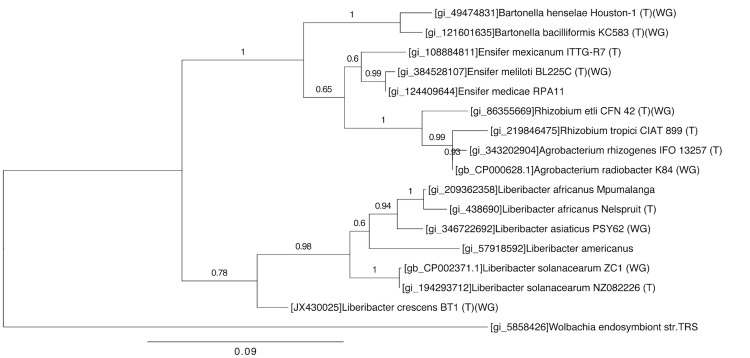
Maximum likelihood phylogenetic tree constructed using 16S rRNA genes of *Liberibacter crescens* BT-1 and related members of the *Alphaproteobacteria*. Branch supports are provided above branches. Sequences were aligned using MUSCLE [12]. Overhanging regions were removed, and the alignment was curated using Gblocks [13]. Phylogeny was determined with PhyML [14] using the GTR substitution model [15] with 500 bootstraps. These tools were accessed through phylogeny.fr [16]. The resultant phylogenetic tree was generated with FigTree [17].

**Figure 2:**
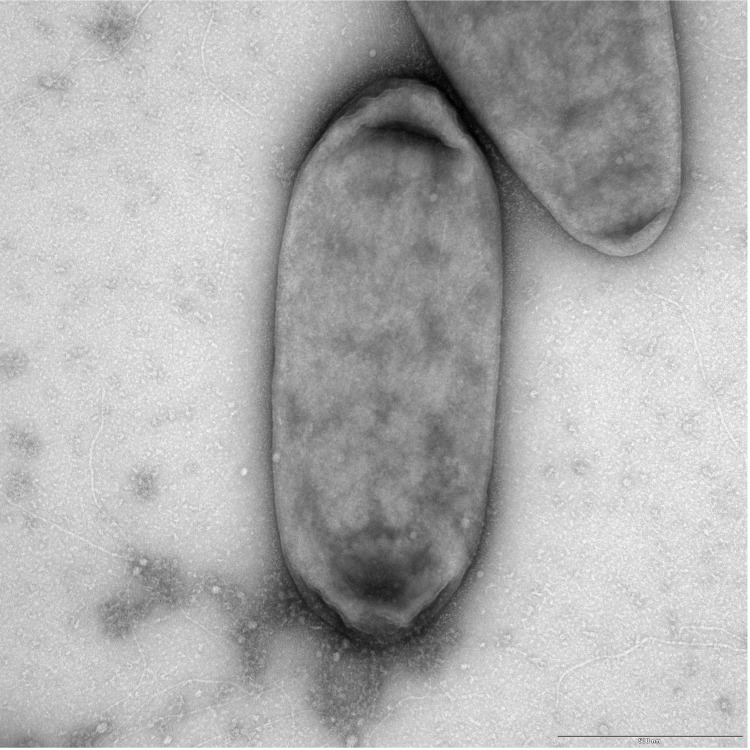
Transmission electron microscopy of *L. crescens* BT-1. Negative stain. Scale bar represents 500 nm.

Figure 1 and Table 1 summarize the phylogenetic position and characteristics of *Liberibacter crescens* BT-1, respectively. Figure 2 shows transmission electron microscopy of *L. crescens* BT-1.

**Table 1:** Classification and general features of *Liberibacter crescens* BT-1 according to the MIGS recommendations [18]

**MIGS ID**	**Property**	**Term**	**Evidence code**^a^
	Current classification	Domain *Bacteria*	TAS [11]
		Phylum *Proteobacteria*	TAS [19]
		Class *Alphaproteobacteria*	TAS [20,21]
		Order *Rhizobiales*	TAS [22]
		Family *Rhizobiaceae*	TAS [23,24]
		Genus *Liberibacter*	TAS [25,26]
		Species *Liberibacter crescens*	
		Type strain *BT-1*	
	Gram stain	negative	TAS [11]
	Cell shape	rod-shaped	TAS [11]
	Motility	nonmotile	IDA
	Sporulation	nonsporulating	IDA
	Temperature range	mesophile	IDA
	Optimum temperature	27**°** C	TAS [11]
	Carbon source	unknown	NAS
	Energy source	unknown	NAS
MIGS-6	Habitat	mountain papaya	TAS [11]
MIGS-6.3	Salinity	unknown	NAS
MIGS-22	Oxygen	aerobic	TAS [11]
MIGS-15	Biotic relationship	endophyte	TAS [11]
MIGS-14	Pathogenicity	none	TAS [11]
MIGS-4	Geographic location	Puerto Rico, USA	NAS
MIGS-5	Sample collection time	1995	NAS
MIGS-4.1	Latitude	18.051944N	NAS
MIGS-4.2	Longitude	67.059722W	NAS
MIGS-4.3	Depth	surface	NAS
MIGS-4.4	Altitude	12 m	NAS

a) Evidence codes - IDA: Inferred from Direct Assay; TAS: Traceable Author Statement (i.e., a direct report exists in the literature); NAS: Non-traceable Author Statement (i.e., not directly observed for the living, isolated sample, but based on a generally accepted property for the species, or anecdotal evidence). These evidence codes are from the Gene Ontology project [27].

## Genome sequencing and annotation

Three sequencing platforms were used to obtain the data necessary to close the genome sequence (Table 2). In addition, other project information and its association with MIGS version 2.0 compliance [32] is provided (Table 2).

**Table 2:** Project information

**MIGS ID**	**Property**	**Term**
MIGS-31	Finishing quality	Finished
MIGS-28	Libraries used	Three libraries: one illumina library, two 454 paired-end libraries (3kb and 8kb insert size), one PacBio standard library
MIGS-29	Sequencing platforms	Illumina GAIIx, 454 GS FLX Titanium, PacBio RS
MIGS-31.2	Fold coverage	6121.2x illumina, 166.9× 454, 31.7× PacBio
MIGS-30	Assemblers	Mira v3.4.0.1 [28]; Amos v3.0.0 [29]; WGS v7.0.1.22 [30]; Velvet v1.2.06 [31]
MIGS-32	Gene calling method	Glimmer [31]
	Genome Database release	
	Genbank ID	CP003789
	Genbank Date of Release	On publication, or August 31, 2013
	GOLD ID	
	Project relevance	Agriculture

### Growth conditions and DNA isolation

The initial culture of BT-1 was obtained in 1995 and was isolated from the peduncle of the tropical Babaco plant, also known as the hybrid mountain papaya (*Carica stipulata* x *C. pubescens*). Babaco was provided by the Lajas Experiment station in Puerto Rico because it showed signs of Papaya bunchy top (PBT), a disease of papaya in the American tropics. The sap of Babaco expressed an extremely high titer of small, rod-shaped bacteria [1].

Despite being fastidious, the bacterium was able to be grown on BM7 media, a modified form of BBM [1,11]. Cells were grown in BM7 liquid culture at 27°C for 4 days in a shaking incubator with a speed of 120 rpm. DNA was extracted using the UltraClean Microbial DNA Isolation Kit and the manufacturer’s protocol (M0-BIO, Carlsbad, CA).

### Genome sequencing and assembly

Sequencing was performed by the Interdisciplinary Center for Biotechnology Research (ICBR) at the University of Florida using the PacBio RS, Illumina GaIIx, and Roche/454 GS/FLX Titanium platforms. The initial draft assembly yielded 5 large (>1,500 bp), non-redundant contigs with an N50 of 379,608bp by combing 831,945 Roche/454 reads (3kb and 8kb insert libraries) at 166.93× coverage, 3,514,850 normalized Illumina reads [33] at 107.95× coverage, and 10,798 corrected PacBio reads [34] at 7.81× coverage by hybrid assembly through the Mira assembler [28]. The resulting maximal base-error rate (<Q40) of the initial assembly was 45 in 50,000.

Contigs were subject to an *Nco*I restriction digest (*in silico*) and mapped to an OpGen optical map of BT-1 using the same enzyme [35]. Optical mapping yielded a circular map of approximately 1.5 Mbp. Misjoined contigs and contig redundancy were indicated by comparison of the scaffold to the optical map of *L. crescens*, and were manually corrected with the CLC Genomics Workbench (CLCbio, Katrinebjerg, Denmark).

Intrascaffold gaps were closed by further passes of the Mira hybrid assembly combining the current scaffold with varying combinations of read data. Omitting certain read technologies at further hybrid assembly iterations allowed more successful assemblies at different points of the genome. Pseudo 454-like paired-end reads were generated from the scaffold to allow very large contigs to be employed in further iterations of Mira hybrid assembly. Pseudo 454-like reads conformed to the 19 kb upper limit of Mira read length and consisted of a 34 kb insert size. Additionally, subsets of the original Illumina paired-end reads and normalized Roche/454 reads were entered into the read pool to avoid problematic reads. Contigs of each hybrid assembly pass were manually corrected for misjoined contigs and combined by Minimus2 [29] to yield a circular genomic sequence.

### Genome annotation

Genome annotation was performed by the Rapid Annotation using Subsystem Technology (RAST) pipeline [36]. RAST employs tRNAscan-SE [37] to identify tRNA genes, Niels Larsen’s "search_for_rnas" (available from the author) to identify rRNA encoding genes, and GLIMMER [38] to identify candidate protein-encoding genes. RAST compares the set of candidate protein-encoding genes to a collection of protein families, referred to as FIGfams [36], in order to correct CDS starting positions and place the genome in a phylogenic context. The candidate protein set was compared to the National Center for Biotechnology Information (NCBI) non-redundant (nr) database, SwissProt database, European Bioinformatics Institute (EBI) phage database, and COG subset of the NCBI Conserved Domain Database (CDD) through the NCBI BLAST suite.

Additionally, predicted proteins were annotated through the Kyoto Encyclopedia of Genes and Genomes (KEGG) automatic annotation server (KAAS). KAAS employs NCBI BLAST to search the KEGG Orthology database [39].

## Genome properties

The genome consists of one circular chromosome of 1,504,659 bp (35.35% GC content). 1,433 genes were predicted, 1,379 of which are protein-coding genes. 1,039 of protein coding genes were assigned to a putative function with the remaining being annotated as hypothetical proteins. The properties and the statistics of the genome are summarized in Tables 3 and 4.

**Table 3:** Nucleotide content and gene count levels of the genome

**Attribute**	**Value**	**% of total^a^**
Genome size (bp)	1,504,659	100
DNA coding region (bp)	1,264,794	84.05
DNA G+C content (bp)	531,980	35.35
Total genes^b^	1433	100
RNA genes	54	3.77
Protein-coding genes	1379	96.23
Genes in paralog clusters	870	63.08
Genes assigned to COGs	857	62.14
Genes assigned Pfam domains	1057	76.65
Genes with signal peptides	84	6.09
Genes with transmembrane helices	327	23.71

a) The total is based on either the size of the genome in base pairs or the total number of protein coding genes in the annotated genome.

b) Does not include pseudogenes or other genes.

**Table 4:** Number of genes associated with the 25 general COG functional categories

**Code**	**Value**	**%age**^a^	**Description**
J	123	14.35	Translation
A	0	0.00	RNA processing and modification
K	21	2.45	Transcription
L	66	7.70	Replication, recombination and repair
B	0	0.00	Chromatin structure and dynamics
D	14	1.63	Cell cycle control, mitosis and meiosis
Y	0	0.00	Nuclear structure
V	12	1.40	Defense mechanisms
T	26	3.03	Signal transduction mechanisms
M	57	6.65	Cell wall/membrane biogenesis
N	31	3.62	Cell motility
Z	0	0.00	Cytoskeleton
W	0	0.00	Extracellular structures
U	19	2.22	Intracellular trafficking and secretion
O	45	5.25	Posttranslational modification, protein turnover, chaperones
C	65	7.58	Energy production and conversion
G	30	3.50	Carbohydrate transport and metabolism
E	96	11.20	Amino acid transport and metabolism
F	41	4.78	Nucleotide transport and metabolism
H	56	6.53	Coenzyme transport and metabolism
I	33	3.85	Lipid transport and metabolism
P	29	3.38	Inorganic ion transport and metabolism
Q	5	0.58	Secondary metabolites biosynthesis, transport and catabolism
R	57	6.65	General function prediction only
S	31	3.62	Function unknown
-	522		Not in COGs

a) The total is based on the total number of COGs in the annotated genome.

## Insights from the genome sequence and comparative genomics

Sequencing of *Liberibacter crescens* BT-1 was conducted to learn why this strain can be cultured while the other *Liberibacter* strains cannot. Also, as BT-1 is not a pathogen of citrus, the BT-1 genome may suggest how *Candidatus* L. asiaticus causes symptoms on citrus while BT-1 does not. Members of the *Liberibacter* genus (*Candidatus*. L. asiaticus, *Candidatus* L. africanus, and *Candidatus*. L. americanus) are known to be the causative agent of Huanglongbing (HLB), commonly called citrus greening, and other HLB-like diseases (*Candidatus* L. solanacearum) [5-7]. However, some members of the *Liberibacter* genus are non-pathogenic, *Candidatus* L. europeaus [8] and *L. crescens* [11].

Although *L. crescens* is currently the only member of the *Liberibacter* genus to be cultured, the sequences of *Candidatus* L. asiaticus and *Candidatus* L. solanacearum are available through NCBI. Comparison of gene function and sequence in BT-1 to *Candidatus* L. asiaticus and *Candidatus* L. solanacearum provided insight to both the virulence and the fastidious nature of the *Liberibacter* genus. Additionally, the *Liberibacter* genus is predicted to be susceptible to bacteriophage insertions, which were also analyzed between the known genomes.

### Sequence comparison of *L. crescens* to *Ca*. L. asiaticus and *Ca*. L. solanacearum

KEGG orthology and RAST automated annotation were the basis of functional comparison of the genes in *L. crescens* to the genes in *Candidatus* L. asiaticus and *Candidatus* L. solanacearum.

Analysis of KEGG orthology uncovered the complete inability of *Candidatus* L. asiaticus and *Candidatus* L. solanacearum to synthesize histidine, tryptophan, and thiamine, as well as a severely reduced ability to produce phenylalanine and tyrosine when compared to *L. crescens*. *Candidatus* L. asiaticus and *Candidatus* L. solanacearum both possess 2 out of the 12 enzymes required for phenylalanine and tyrosine biosynthesis. To compensate, all three species possess a general L-amino acid ATP-binding cassette (ABC) transporter. ABC transporters are known to be associated with nutrient uptake, drug resistance, and virulence [40,41]. Also, *Candidatus* L. asiaticus and *Candidatus* L. solanacearum possess a thiamine ABC transporter not found in *L. crescens*, presumably to compensate for the inability to synthesize thiamine. These deficiencies provide insight into the metabolic requirements of the uncultured *Liberibacter* species.

Furthermore, KEGG orthology and RAST annotation indicate the presence of a zinc ABC transporter in all three species. Transporters of metal ions have been shown to play a role in bacterial virulence, including ABC transporters of iron, zinc, and manganese [42,43]. Although the zinc transporter was located in *L. crescens* through RAST annotation, it was not detected by KEGG orthology. This discrepancy is attributed to a low sequence similarity between the protein components of the zinc ABC transporter (ZnuA, ZnuB, ZnuC) in *L. crescens* compared to *Candidatus* L. asiaticus and *Ca*. L. solanacearum, at 43.6%, 55.3%, and 48.5% average similarity for each component, respectively (Table 5). In contrast, the similarity of each component between *Candidatus* L. asiaticus and *Candidatus* L. solanacearum is 78.6%, 93.0%, and 92.2% respectively (Table 5). Sequence similarity was determined through sequence alignment using the EMBOSS Water tool [44] and the EBLOSUM62 scoring matrix. This variation in zinc ABC transport proteins may contribute to the virulence of the *Liberibacter* genus.

**Table 5:** Species similarity of zinc ABC transporter components

	**ZnuA**	**ZnuB**	**ZnuC**
*L. crescens* to *Candidatus* L. asiaticus	43.1%	55.2%	46.9%
*L. crescens* to *Candidatus* L. solanacearum	44.0%	55.4%	50.0%
			
**Average**	43.6%	55.3%	48.5%
*Ca*. L. asiaticus to *Ca*. L. solanacearum	78.6%	93.0%	92.2%

Also present in *L. crescens*, but not in *Candidatus* L. asiaticus and *Candidatus* L. solanacearum, is a twin-arginine translocation (Tat) protein export pathway and an additional iron ABC transporter. The significance of these two transporters is not currently known, but their existence may explain why *L. crescens*, is less fastidious than *Candidatus* L. asiaticus and *Candidatus* L. solanacearum.

Present in *Candidatus* L. asiaticus and *Candidatus* L. solanacearum, but not in *L. crescens,* are several components of a fimbrial low-molecular-weight protein (flp) pilus system. These pili are involved in tight adherence and are encoded by the Tad family proteins [7]. Diversity in the flp pilus operon is predicted to contribute to variation in virulence among pathogenic species [45-48], and provides further insight to the virulence of the *Liberibacter* genus.

### Phages in the genomes of *Candidatus* L. asiaticus and *L. crescens*

Recently, two prophages, SC1 and SC2, were found to exist in tandem in *Candidatus* L. asiaticus through DNA isolation from diseased citrus phloem and an insect vector of the family *Psyllidae* [10]. *Candidatus* L. solanacearum is known to host two prophage regions as well, not in tandem, with one region maintaining a high degree of similarity with the prophage regions in *Candidatus* L. asiaticus and the other containing a small segment with lower similarity [7]. Two putative prophages were found in the *L. crescens* genome through the use of the Prophage Finder tool [49], the Phage_Finder [50] tool, and the methods described in Casjens et al (2003).

Prophage boundary identification is an inexact process due to the diversity of bacteriophages, and is made even more difficult by the possibility of evolutionary decay of prophages that do not enter a lytic cycle. Additionally, prophage boundaries are indicated by a multitude of factors, but not defined by any particular criteria. Position of nearby tRNAs close to the predicted prophage region may be indicative of a boundary, as tRNAs are often sites of phage insertion [50]. A sharp shift in G+C content at the predicted prophage region may also indicate the range of phage insertion, but only if the phage G+C content differs dramatically from the host. Certain genes are unique to phage genomes, and non-phage genes were not typically found to be present between phage genes in an inserted phage. From a genomic standpoint, prophage regions are also indicated by regions not present in closely related species, as well as long strings of unidentified proteins in similar orientation [51].

**Figure 3:**
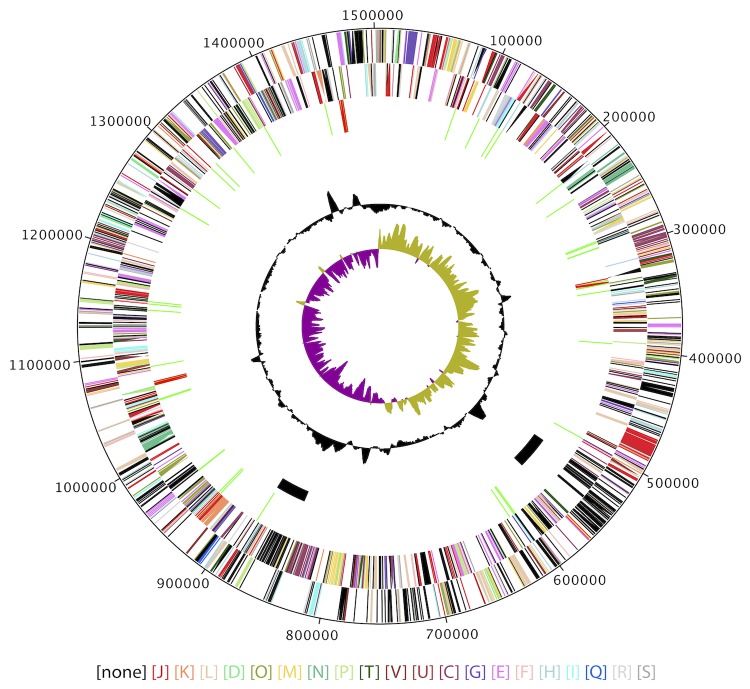
Circular genomic map of *L. crescens* BT-1. From outside to the center: Genes on forward strand (colored by labeled COG categories), genes on reverse strand (colored by labeled COG categories), RNA genes (tRNA green, rRNA red), putative prophage regions, GC content, GC skew.

**Figure 4:**
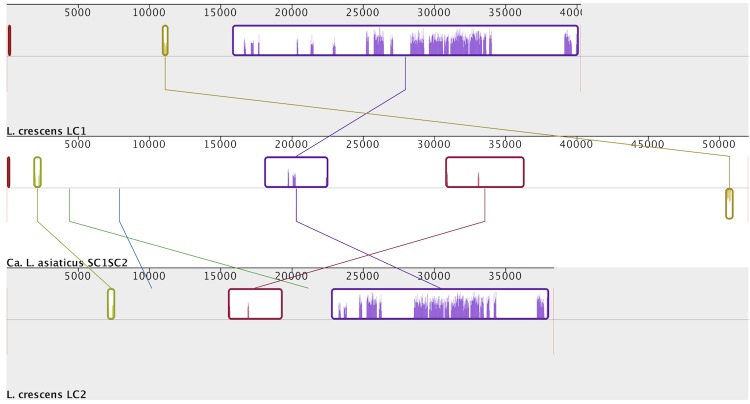
Whole sequence alignment of phage regions between *L. crescens* and *Candidatus* *L. asiaticus*. The two prophage regions *Candidatus* L. solanacearum are homologous, and both share higher similarity with the prophage region in *Candidatus* L. asiaticus. Graphical representation obtained through Mauve [53].

**Figure 5:**
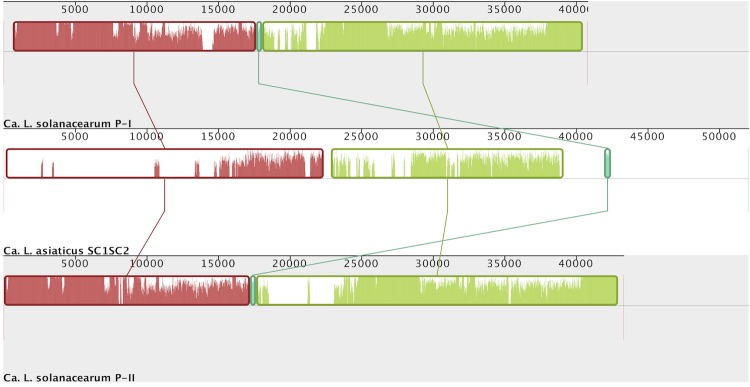
*Candidatus* L. solanacearum and *Candidatus* L. asiaticus. Signifies that prophages in *L. crescens* are not homologous to each other or to the tandem prophage region in *Candidatus* L. asiaticus.

From the above criteria, the locations and boundaries of two prophages in *L. crescens* were predicted to extend from base pair 523,789-564,039 in prophage LC1 and from base pair 848,435-886,798 in prophage LC2. Unlike the two prophages in *Candidatus* L. asiaticus, the prophages in *L. crescens* were not homologues, sharing only short (<1,000 bp) regions of moderate similarity, determined through Wise2 alignment [52]. Additionally, the prophages in *L. crescens* were not found in *Candidatus* L. asiaticus. Homology was inferred through alignment by the progressiveMauve algorithm [53] (Figures 3-5). While the SC1 phage in *Candidatus* L. asiaticus is known to enter a lytic cycle in the phloem of citrus, the lifecycles of the prophages in *L. crescens* have yet to be explored experimentally [10].

Interestingly, the same zinc ABC transporter mentioned above is present in the LC2 region*.* Prophage insertions have been known to add functions to hosts, making the host more competitive [54]. In addition to metabolic variation, the differences in extra-chromosomal genomic content between species of the *Liberibacter* genus may also be indicative of the virulence and fastidious nature of the genus.

## Conclusion

*Liberibacter crescens* BT-1 is the first member of the *Liberibacter* genus to be cultured. The complete genome sequences of *Candidatus* L. asiaticus and *Candidatus* L. solanacearum have been determined through isolation from the disease vectors [7,9], but any attempt to culture these species typically depends on employing a co-culture with insect or plant host cells [5]. Genomic sequencing of *L. crescens* BT-1 was performed in an attempt to find possible indications for virulence in *Candidatus* L. asiaticus and *Candidatus* L. solanacearum, as well as an explanation for the fastidious nature of these pathogens.

Assembly of *L. crescens* yielded a complete genome containing two predicted prophages. Sequence comparison of *Candidatus* L. asiaticus to *L. crescens* indicated that the species are 75.5% similar [11]. However, the prophage regions are not homologous. Sequencing and analysis of the *L. crescens* genome provided insight to the metabolic requirements of *Candidatus* L. asiaticus, which appears to lack the ability to synthesize thiamine and several essential amino acids. Less is known about the virulence of *Candidatus* L. asiaticus, although bacteriophages have also been known to add virulence to an otherwise non-pathogenic bacterium [54]. Further genomic analysis indicated that virulence in *Candidatus* L. asiaticus could also be due to a zinc ABC transporter. While the sequencing of *L. crescens* gave much insight into the *Liberibacter* genus, further experiments must be conducted to verify these predictions.
